# Posterior hemivertebra resection with unilateral instrumented fusion in children less than 10 years old: preliminary results at minimum 5-year follow-up

**DOI:** 10.1186/s13018-018-0946-3

**Published:** 2018-09-20

**Authors:** Xuhong Xue, Sheng Zhao

**Affiliations:** Department of Orthopedics, The Second Hospital of Shanxi Medical University, Taiyuan, No. 382 Wuyi Road, Taiyuan, 030001 Shanxi People’s Republic of China

**Keywords:** Hemivertebrae, Unilateral fusion, Hemivertebrectomy, Congenital scoliosis

## Abstract

**Background:**

The main treatment for congenital hemivertebra is posterior hemivertebrectomy with bilateral transpedicular fixation. To date, studies describing posterior unilateral fusion are few, especially in younger children. The modified method by posterior hemivertebrectomy combined with unilateral transpedicular instrumentation and fusion was described. The purpose was to present the clinical and radiological outcome of children less than 10 years treated for congenital scoliosis with posterior hemivertebrectomy and unilateral instrumented fusion.

**Methods:**

A study of 43 consecutive patients through Jan. 2006 to Mar. 2013 for hemivertebrae in children less than 10 years was performed. Patients undergoing hemivertebrectomy and posterior convex short-segment fusion, which had been followed up for at least 60 months, were included. Coronal main curve, kyphosis, T1-S1 height, fused vertebra height, and concave height were measured at preoperation, immediate postoperation, and final follow-up. The outcome and efficacy of the correction provided and growth of the non-fused concave side of the spine was investigated.

**Results:**

The average follow-up period was 73.88 ± 16.77 months. The mean Cobb angle of the coronal curve was improved from 46.1 to 8.1° (correction rate 82.4%). At final follow-up, there was 7.8% loss of correction. The average concave height, fusion segment height, and T1-S1 height were 60.1 ± 19.7 mm, 56.9 ± 22.9 mm, and 326.6 ± 64.5 mm in immediate postoperation, which improved to 73.1 ± 23.7 mm, 71.2 ± 22.0 mm, and 388.7 ± 78.9 mm at the last follow-up. These parameters were significantly different between the immediate postoperation and at final follow-up. The rate of reoperation was 9.3% (4/43), mainly in PJK and curve progression after surgery.

**Conclusions:**

Despite with some complications, posterior hemivertebrectomy and unilateral instrumented fusion are commendable procedures. We concluded that it is a simple, secure, reliable, less-invasive, and well-tolerated technique that can successfully resolve this kind of congenital scoliosis in children.

## Background

Congenital scoliosis encompasses a continuous bending of spinal deformities, which result from the localized imbalance in the longitudinal growth of the spine caused by asymmetrical development of one or more vertebras [1]. It may be classified into failures of formation, failures of segmentation, or mixed deformities [2]. The most common anomaly caused by failure of formation is the hemivertebra. Based on pathological features, hemivertebra has been described to three types including (1) the fully segmented, (2) the semi-segmented, and (3) the incarcerated types [3]. Incarcerated hemivertebra and a balanced trunk usually have a benign result, while non-incarcerated hemivertebra has a normal-growth plate leading to progression of a wedge-shaped deformity. Scoliosis caused by non-incarcerated hemivertebra is more rigid than other types, which is difficult to correct by conservative treatment such as back brace and cast, which often requires surgery for correction [4, 5].

Posterior or anterior convex growth arrest (CGA) has been used to the treatment of early-onset congenital scoliosis [6]. Although successful results have been reported with the technique, the failure rates ranging from 8 to 21% have also been reported [7]. Several drawbacks of the classic CGA have also been described. On the one hand, the need for an additional anterior procedure has been one of the most worrisome drawbacks of the procedure. On the other hand, some problems such as being unable to obtain acute correction and unpredictability of gradual correction after surgery have been reasons of failure in CGA [8].

To negate the previously mentioned drawbacks, the technique has been modified. We applied pedicle screws through the posterior approach on the convex short-segment instrumentation aiming to control convex growth. Meanwhile, hemivertebra resection via the posterior approach was adopted aiming to achieve acute correction. Personalized hemivertebra excision was introduced to balance the growth potential in both sides of the spine. It can achieve convex growth arrest and preserve concave growth potential at the same time. The outcome and efficacy of this technique has been investigated in the present study.

## Methods

With the approval of the institution’s Institutional Review Board, 43 consecutive patients with hemivertebrae younger than 10 years of age were treated using hemivertebrectomy and posterior convex short-segment fusion through Jan. 2006 to Mar. 2013 at a single spine center. They had been followed up for at least 60 months. There were 23 boys and 20 girls. There were 22 fully segmented and 21 semi-segmented hemivertebra. The hemivertebra was located within the thoracic spine (T1–T11) in 27 cases, within the thoracolumbar region (T12–L1) in seven cases, and within the lumbar spine (L2–L5) in 11 cases. Three patients had contralateral bar formation and synostosis of the ribs. Most of abnormal vertebra was located at the right side, only three cases within the left side. We used a 4.5-mm rod with polyaxial pediatric posterior instrumentation screws of 4.0-mm diameter in six patients whose ages were below 3 years, and we used a 5.5-mm rod with a 4.75- or 5.0-mm-diameter pediatric posterior instrumentation screws in the remaining 37 patients (Bonovo Orthopedics, Inc).

Preoperative evaluation included a thorough neuromuscular examination, routine radiographs, 3-dimensional computed tomography scan, and magnetic resonance imaging due to increased incidence of intraspinal abnormalities associated with congenital scoliosis. Cardiovascular and urogenital examinations were performed to detect congenital heart diseases and abnormalities of the renal system. None of the patients had undergone a prior operation. None had a neurologic deficit or cord anomaly. Surgery was indicated by proved or expected deterioration of the deformity.

**Fig. 1:**
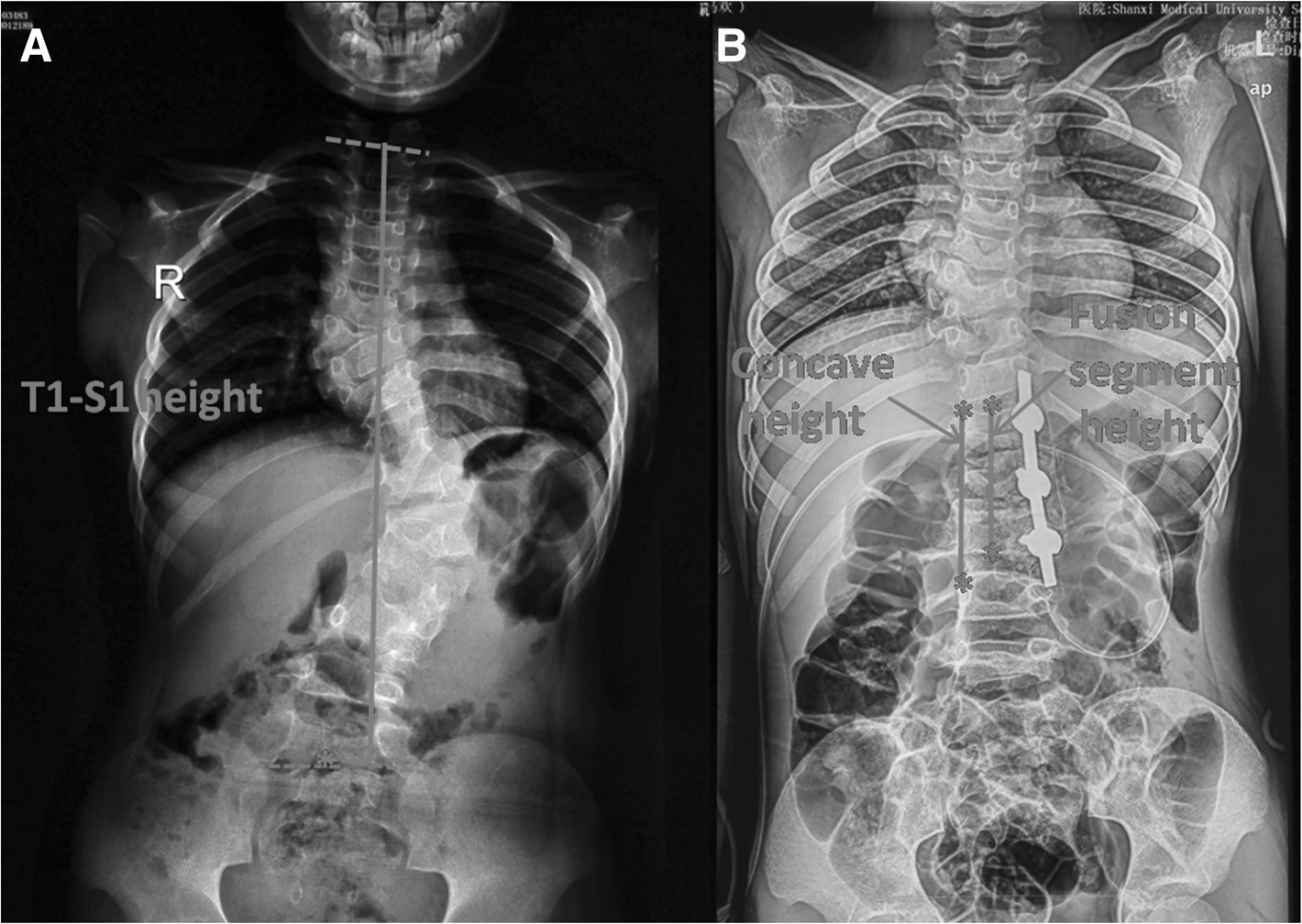
T1-S1 height: the vertical distance from the midpoint of the superior endplate of T1 to the midpoint of the superior endplate of S1 (**a**); fusion segment height: vertical distance from the midpoint of the superior endplate of upper UIV to the midpoint of the superior endplate of LIV; concave height: the height between the concave side pedicles of the upper and lower end vertebra of the main curve (**b**)

Preoperation, immediate postoperation, and final follow-up standing posteroanterior and lateral radiograms were evaluated. Coronal main curve was recorded for the instrumented segment. Global thoracic kyphosis was measured between T2 and T12 on a sagittal plane. These values were compared preoperatively, postoperatively, and at last follow-up. The height between T1 and S1 was determined by the vertical distance from the midpoint of the superior endplate of T1 to the midpoint of the superior endplate of S1 (Fig. 1a). The height of fusion segment was determined by the vertical distance from the midpoint of the superior endplate of the upper instrumented vertebra (UIV) to the midpoint of the superior endplate of the lowest instrumented vertebra (LIV) (Fig. 1b). The height between the concave side pedicles of the upper- and lower-end vertebra of the main curve was also determined and recorded as the concave height (Fig. 1b). These measurements of the height on the radiograph are done digitally in early postoperative and at final follow-up X-rays. Operative reports were reviewed to determine the presence of any intraoperative complication. Medical records were reviewed to identify any complication in the perioperative and follow-up periods. Measurements were taken from standing long-cassette anterior-posterior and lateral radiographs.

### Surgical technique

**Fig. 2:**
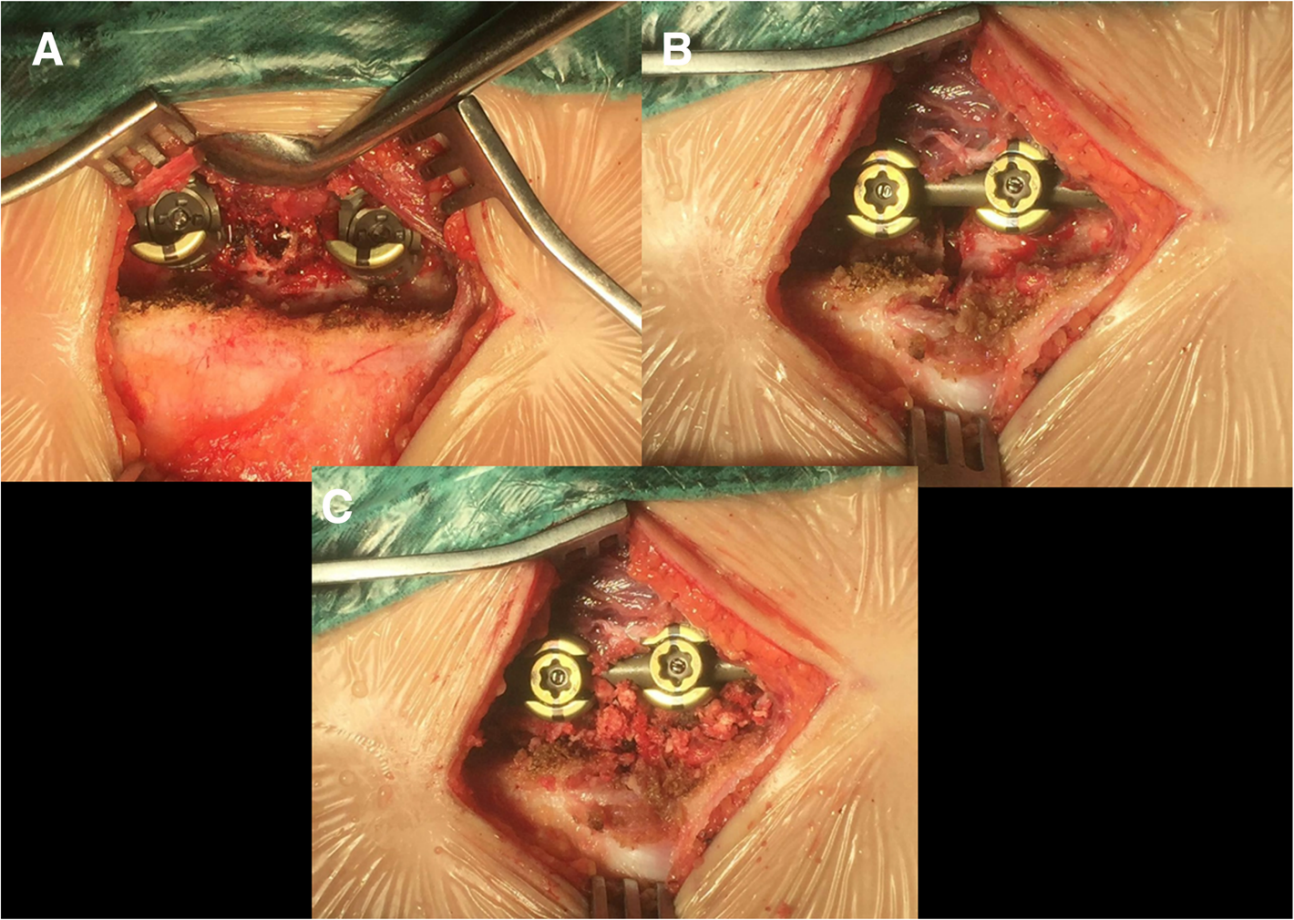
Interoperation image**:** convex side only to expose the hemivertebra, and unilateral pedicle screws were inserted into the vertebra using the funnel technique (**a**). Hemivertebra resection: the vertebral end plates of the convex side were excised (**b**). Gradual compression until the gap was closed. Bone graft for fusion of the posterior elements (**c**)

The patients were positioned prone on the operating table. A standard midline skin incision was used, and subperiosteal dissection was performed on the convex side only to expose the hemivertebra and the vertebrae just above and below, including the lamina, transverse processes, and facet joints. Fluoroscopy was used for the determination of instrumentation levels. Unilateral pedicle screws were inserted into the vertebra above and the vertebra below using the funnel technique described by Viau et al. [9]. Briefly speaking, the posterior cortex of the lamina overlying the top of the pedicle is removed by a rongeur to make a 6–8-mm-diameter opening in the cortex. The cancellous isthmus of the pedicle is directly visualized by removing the cancellous bone from the upper part of the pedicle with a small curette. After further removal of the cancellous bone, enlargement of the pedicle funnel leads the curette into the upper part of the pedicle isthmus. The cortical margins of the upper part of the pedicle then act as a funnel to permit safe insertion of the pedicle probe through the pedicle isthmus. Firstly, careful probing of the pedicle is then performed with the 2-mm probe, and then, if the pedicle inner diameter allows it, with the larger pedicle probe. At last, a ball-tip probe is used to inspect the depth of the opening as well as all four walls of the pedicle for a perforation (Fig. 2a).

**Fig. 3:**
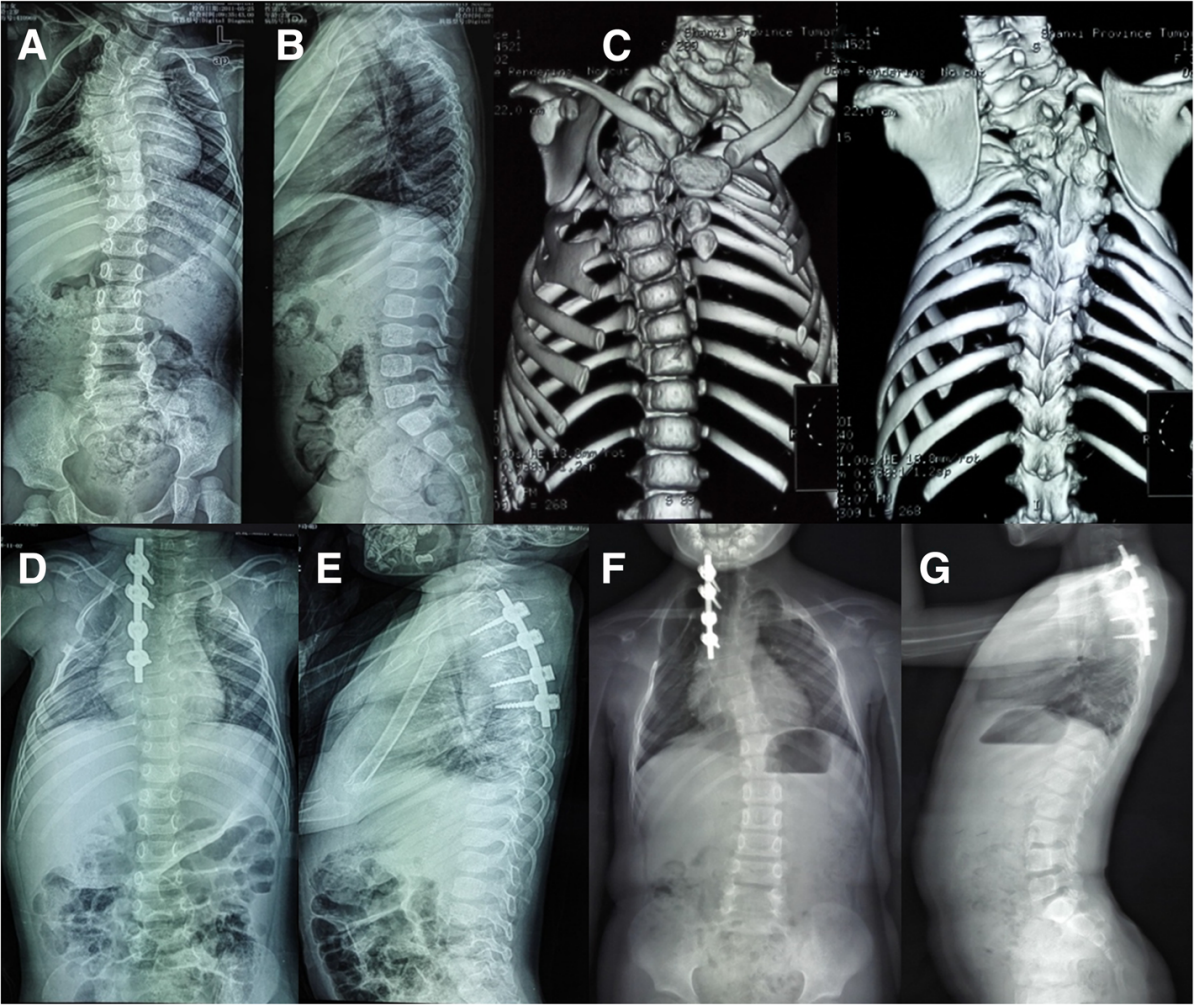
A 2 years and 4 months old girl with congenital scoliosis. Radiographs and 3D-computed tomography images obtained preoperatively (**a**, **b**, **c**), postoperatively (**d**, **e**), and at the latest follow-up visit, 60 months later (**f**, **g**)

**Fig. 4:**
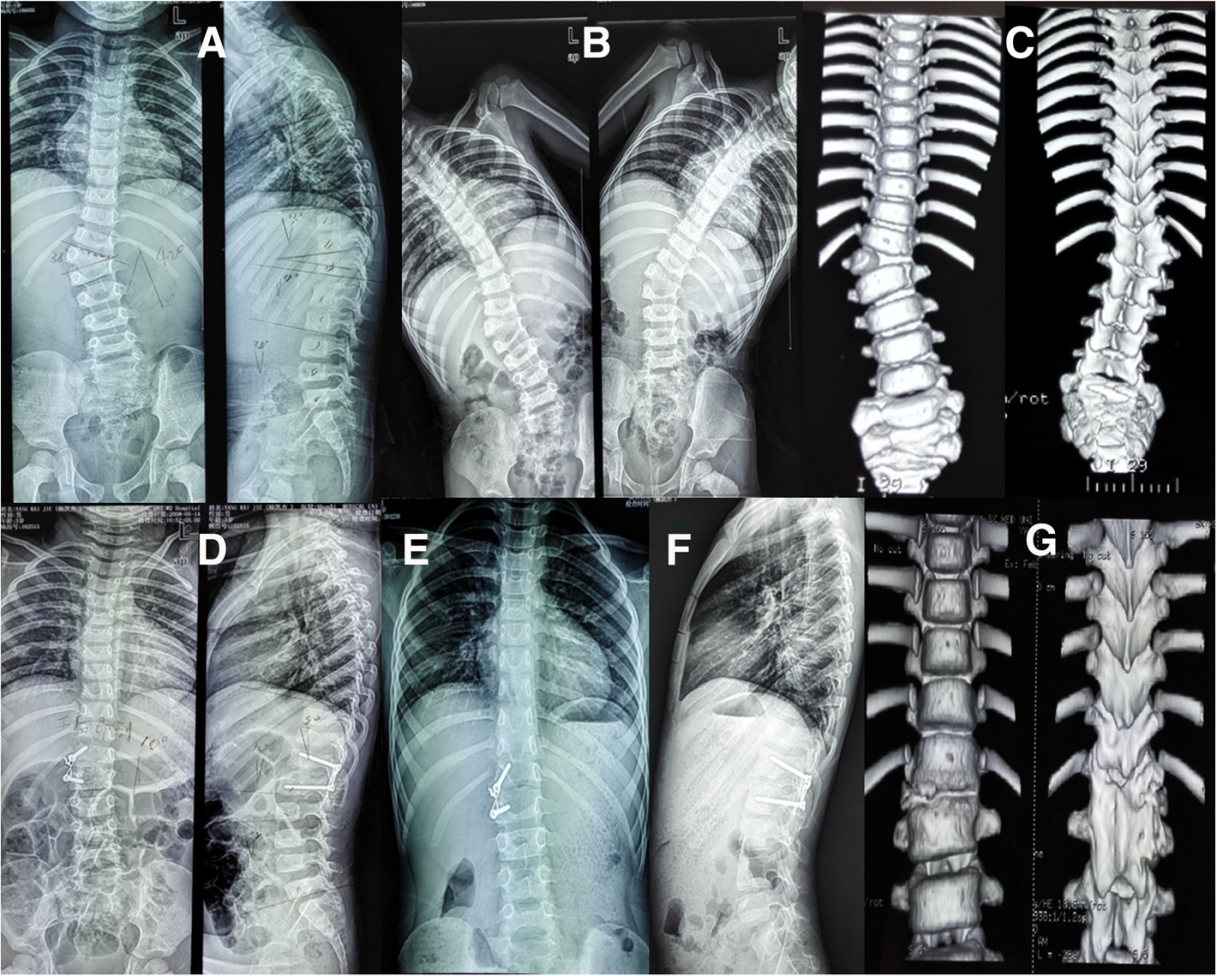
A 2 years 9 months old girl with T12 hemivertebra. Radiograph images and 3D-computed tomography images obtained preoperatively (**a**–**c**), postoperatively (**d**), and at the latest follow-up visit, 69 months later (**e**, **f**, **g**)

The posterior parts of the pedicle were removed; inferior half of the lamina above was excised including the inferior articular process, and then, the superior articular process of the hemivertebra was excised (Fig. 2b). This was followed by excision of the inferior articular process of the hemivertebra and the superior articular process of the vertebra below. If in the thoracic spine, the rib head was also removed. The spinal cord and the nerve roots were identified and protected, as well as the pleura. Epidural veins were cauterized by bipolar cautery to allow clear visualization. The remnants of the vertebral body of the hemivertebra and the adjacent disks were removed. The vertebral end plates of the convex side were debrided down to the bone, and the contralateral end plate was retained (Fig. 2c). The concave side was not exposed as continued spinal growth on this side will improve the correction in 40 patients. Subsequently, a pre-contoured rod was connected to the screws on the convex side. Gradual compression was applied until the gap was closed. Bones retrieved during osteotomy were used as graft material for fusion of the posterior elements. In cases with contralateral bar formation and multiple rib anomalies, unilateral exposure is not sufficient and bilateral exposure is necessary to cut off the bar or synostosed rib heads. Three cases involved a double hemivertebra or concave bar and multiple rib anomalies, which required extension of the fusion to more segments (Fig. 3). In one patient aged 2.8 years, no appropriately sized pedicle screw was available; therefore, 3.0-mm, cortical screws combined with wires were used as an alternative (Fig. 4). For lumbar or lumbosacral hemiveterbra, unilateral short-segment instrumentation combined with hemivertebra resection was also very applicable.

After surgery, all patients were fitted with a rigid brace to protect the instrumentation for at least 6 months.

### Statistics

Paired *t* test was used to analyze the difference of coronal curve angle, thoracic kyphosis, segmental kyphosis, T1-S1 height, fusion segment height, and concave height at preoperation, postoperation, and final follow-up. SPSS version 17.0 (SPSS Inc., Chicago, IL) was used in all statistical analyses. The differences with a *P* value less than 0.05 were considered as statistically significant.

## Results

**Fig. 5:**
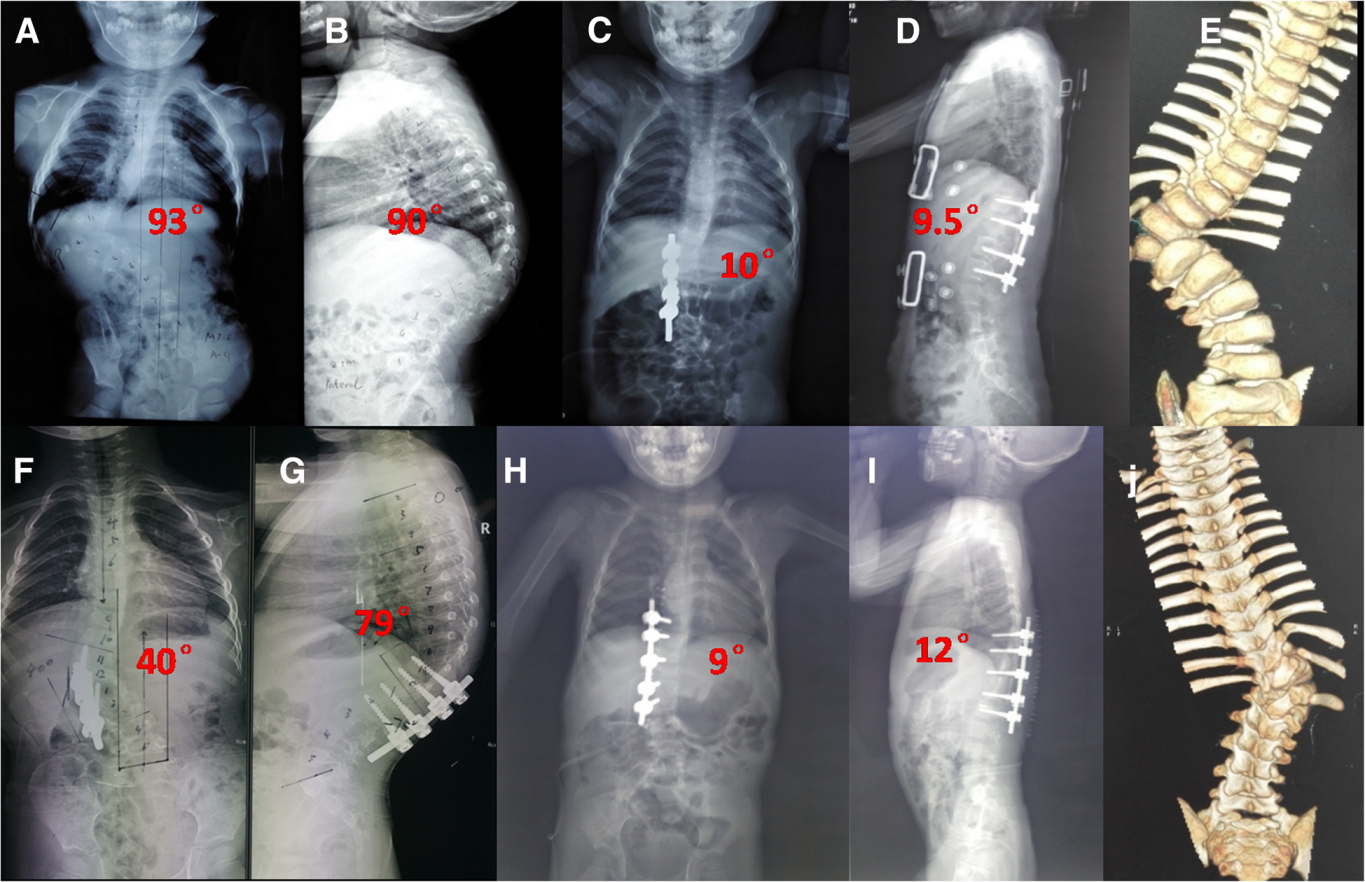
A 2.5-year-old boy with T12 hemivertebra. Radiograph images and 3D-computed tomography images obtained preoperatively (**a**, **b**, **e**, **j**), postoperatively (**c**, **d**), and at the latest follow-up visit, 12 months later (**f**, **g**) and revision surgery (**h**, **i**)

Of the 43 patients, 10 had rib anomalies, 3 had concave unsegmented bar, 1 had congenital cardiac anomalies, and 6 had intraspinal anomalies. No intraoperative complications were noticed. There were no neurologic complications. No implant failure was found at the final radiographic evaluations. Four patients experienced reoperation, and the overall rate of reoperation was 9.3%; demographic data are summarized in Table 1. The reasons of reoperation included two proximal junctional kyphosis (Fig. 5) and two curve progressions in the distal junction. In total, the average follow-up period was 73.88 ± 16.77 months (range, 60 to 112 months). Average age of the patients at surgery was 6 years and 7 months (range, 2 to 10 years). There were no major vascular or neurological complications.

**Table 1:** Patients’ demographic data in revision surgery

Patients	Sex	Age	Abnormality	Association anomalies	Revision reason	Initial surgery	Duration time (months)	Revision surgery	Final F/U (months)
1	M	8	HV:T5; Unsegment T3–7	Sprengel deformity	PJK	T5 HV resection with T4–7 convex fusion	11	T2 PSO with T1–3 convex fusion	112
2	M	2	HV:T12	–	PJK due to T11 pedicle fracture	T12 HV resection with T10-L2 convex fusion	12	T11 Y-shape osteotomy with T9-L2 convex fusion	45
3	F	7	HV:T6; Unsegment T5–9	Fused rib in concave (9–10)	Curve progression in distal junction	T6 HV resection with T5–7 convex fusion	27	T7 PSO with T5–10 convex fusion	80
4	M	5	HV:T6; BF:T10; Unsegment L2–3	–	Curve progression	T6 HV resection with T3–9 convex fusion	78	Bilateral fusion from T3 to L3	90

*F* female, *M* male, *HV* hemivertebra, *BF* butterfly vertebra

When all the patients were evaluated together, the average coronal curve magnitude was 46.1 ± 19.6° (range, 21 to 93°) preoperatively, 8.1 ± 9.6° (range, 0 to 37°) immediate postoperatively, and 11.7 ± 12.2° (3 to 49°) at last follow-up. The difference between the preoperative and early postoperative main curve Cobb angle measurements was significant (*P* = 0.000). At the latest follow-up, there was 7.8% (3.6°) loss of correction. The global thoracic kyphosis was 35.5 ± 21.7° (range, 18 to 72°) preoperatively, 34.9 ± 7.7° (15 to 41.5°) immediate postoperatively, and 37.6 ± 7.5° (19 to 44°) at last follow-up. The segmental kyphosis was 23.3 ± 27.2° (3 to 90°) preoperatively, 10.1 ± 3.1° (9 to 15°) immediate postoperatively, and 7.4 ± 7.2° (1 to 22°) at the last follow-up assessment resulting in a mean improvement of 13.2° postoperatively, and this improvement continued at the latest follow-up with a mean increase of 2.7°(Table 2).

**Table 2:** Summary of radiographic parameters preoperatively and postoperatively

	Preoperation	Postoperation	*P* value
Coronal curve cobb(°)	46.1 ± 19.6	8.1 ± 9.6	0.000
Thoracic kyphosis(°)	35.5 ± 21.7	34.9 ± 7.7	0.951
Segmental kyphosis(°)	23.3 ± 27.2	10.1 ± 3.1	0.007
T1-S1 height(mm)	322.9 ± 63.4	356.9 ± 60.3	0.088

The average concave height was 60.1 ± 19.7 mm in the immediate postoperative period and 73.1 ± 23.7 mm at last follow-up. There was a significant difference between immediate postoperative and at last follow-up measurements (*P* = 0.003). The average fusion segment height was 56.9 ± 22.9 mm in the immediate postoperative period and 71.2 ± 22.0 mm at last follow-up (*P* = 0.002). The average T1-S1 height was 326.6 ± 64.5 mm in the immediate postoperative period and 388.7 ± 78.9 mm at last follow-up (*P* = 0.001) (Table 3). The average fusion segment was 2.9 ± 1.8 levels (2–6 levels).

**Table 3:** Summary of radiographic parameters postoperatively and at the last follow-up

	Postoperation	Last follow-up	*P* value
Coronal curve cobb(°)	8.1 ± 9.6	11.7 ± 12.2	0.031
Thoracic kyphosis(°)	36.7 ± 8.3	37.6 ± 7.5	0.523
Segmental kyphosis(°)	10.1 ± 3.1	7.4 ± 7.2	0.346
T1-S1 height (mm)	326.6 ± 64.5	388.7 ± 78.9	0.001
Concave height (mm)	60.1 ± 19.7	73.1 ± 23.7	0.003
Fusion segment height (mm)	56.9 ± 22.9	71.2 ± 22.0	0.002

## Discussion

The natural history of congenital scoliosis is still unknown. There is no treatment algorithm because of the variability of surgical solutions. The coronal and sagittal deformities, as well as the patient age and type and location of the anomaly, should be taken into consideration. As the most frequent cause of congenital scoliosis, most untreated fully segmented and semi-segmented non-incarcerated hemivertebra will progress and create a wedge-shaped deformity during the spinal growth period [10]. In this situation, the conditions of most patients cannot be controlled with nonsurgical treatments like wearing orthosis. Thereby, most spine surgeon had reached a consensus on surgical treatment for congenital scoliosis due to hemivertebra.

At present, the recommended surgical options mainly include in situ fusion, convex growth arrest, and hemivertebra excision via a combined anterior and posterior approach in one or two stages [2, 11–13]. Currently, the one-stage posterior hemivertebra resection combined with bilateral transpedicular screw instrumentation has become the commonly adopted procedure for the correction of congenital scoliosis. The use of pedicle screws is a powerful method which allows excellent deformity correction in the coronal and sagittal planes, and is safe, even in very young children [10]. The pedicle screw instrumentation can effectively transmit the correction torque to the anterior and middle columns of the spine, increasing the compressive resistance of the anterior and middle columns [14]. Thereby, it helps retard the growth of vertebrae and prevents the crankshaft phenomenon.

Despite the major improvements in the treatment of congenital scoliosis due to hemivertebra, described, simple, and less-invasive methods were more acceptable and popular. The posterior unilateral transpedicular screw fixation could be an advisable alternative to further minimize the trauma in children and decrease the growth arrest of the concave side. Moreover, short-segment fusion can be performed to preserve more growing segments and motor units. Most of the concave pedicle was anomaly and tiny in malformed vertebra; thus, pedicle screw placement is very difficult, having to extend the instrument segment if bilateral transpedicular screw fixation was selected.

In the present study, the mean correction rates of the segmental curve and segmental kyphosis were 82.4% and 56.2%, respectively. Deformities were satisfactorily corrected, and there were no significant losses during the follow-up, suggesting that unilateral transpedicular fixation can provide sufficient force for correction in children. These correction rates are in accordance with previous reports of posterior bilateral fixation describing similar case series [13, 15]. For these patients with scoliosis progression in follow-up, including two curve progression in the proximal junction and two cases in the distal junction, insufficient fusion levels and complex congenital scoliosis may be the main cause.

Unilateral short-segment fixation requires pedicle screw strength. Pedicle fracture, which requires revision surgery, is the most common complication reported in previous studies [16, 17]. In our series, only one patient experienced pedicle fracture that was revised. The patient with severe kyphoscoliosis due to T12 hemivertebrea shows proximal junction kyphosis in 7 months after initial hemivertebrectomy and unilateral fusion. The rigid curve, poor flexibility for kyphoscoliosis, and poor adherence for brace may be the cause. Many studies have confirmed that pedicle fractures are related to the surgical technique rather than to the weakness of the pedicle [15]. Therefore, we adopted the funnel technique in all cases to guarantee accuracy of the pedicle screw placement. Most important of all, the use of a brace post operation is very necessary, not only to protect the instrumentation, but also to maintain further correction. We recommend the minimum 6-month duration of brace use, even after the bone fusion is complete.

The safety of the pedicle screws in the pediatric spine and their effect on vertebral growth have been the topic of controversy in the literature, as some experimental studies have shown growth retardation secondary to pedicle screw instrumentation in immature animals [18, 19]. However, prior clinical studies have shown no growth-retardation effect of the pedicle screws in the pediatric spine [20]. A recent study by Xue et al. has also observed no negative effects of the pedicle screw instrumentation on vertebral bodies’ growth. No spinal stenosis existed in an average of 7 years after instrumentation on CT axial images. In their study, 35 patients less than 7 years were consisted undergoing pedicle screw instrumentation [21].

The limitation of this study was that it is a retrospectively reviewed study with a small number of patients included; more patients are needed in the future. The other drawback was that all patients have been followed-up for at least 60 months, which are very young and far from mature bone; thus, continuous follow-up is needed in the future. In addition, this study does not contain results about life quality in the follow-up. Further trials about life quality, especially spinal mobilization and mental health status, are needed to be evaluated in the future.

## Conclusions

Despite having some complications, posterior hemivertebrectomy and unilateral instrumented fusion are commendable procedures for early correction in young children. The safety and efficacy of the correction were proved by radiographic measurements. The growth of the non-fused concave side of the spine was observed. We concluded that it is a simple, secure, reliable, less-invasive, and well-tolerated technique that can successfully resolve this kind of congenital scoliosis in children.

## Abbreviations

CGA: Convex growth arrest
LIV: Lowest instrumented vertebra
PJK: Proximal junction kyphosis
MRI: Magnetic resonance imaging
SPSS: Statistic Package for Social Science
CT: Computed tomography
UIV: Upper instrumented vertebra
